# Tyrosine Kinase Receptor ErbB4 in Advillin-Positive Neurons Contributes to Inflammatory Pain Hypersensitivity in Mouse DRG

**DOI:** 10.14336/AD.2023.1110

**Published:** 2023-11-20

**Authors:** Zhongxin Guo, Qingyun Huang, Wei Zhang, Kaiyue Shi, Jie Yuan, Shuya Qi, Bingyan Wang, Kuotao Li, Shuntang Li, Jiangu Gong, Xuechao Jing, Yuanyuan Liu, Guohe Tan

**Affiliations:** ^1^Institute of Neuroscience and Guangxi Key Laboratory of Brain Science, Department of Human Anatomy, School of Basic Medical Sciences, Guangxi Medical University, Nanning, Guangxi, China.; ^2^Key Laboratory of Longevity and Aging-related Diseases of Chinese Ministry of Education, Guangxi Health Commission Key Laboratory of Basic Research on Brain Function and Disease, Nanning, Guangxi, China.; ^3^Guangxi Key Laboratory of Regenerative Medicine, Collaborative Innovation Centre of Regenerative Medicine and Medical BioResource Development and Application Co-constructed by the Province and Ministry, Nanning, Guangxi, China.; ^4^China-ASEAN Research Center for Innovation and Development in Brain Science, Nanning, Guangxi, China

**Keywords:** ErbB4, dorsal root ganglion, advillin, inflammatory pain, pain hypersensitivity

## Abstract

Inflammatory pain is a common type of pathological pain. Although the dorsal root ganglion (DRG) is key to pathogenesis of inflammatory pain, the underlying specific molecular and cellular mechanisms remain unclear. In this study, we used mouse models of acute or chronic inflammatory pain, induced by formalin or complete Freund’ s adjuvant (CFA), respectively, to explore whether tyrosine kinase receptor ErbB4 participates in the pathogenesis of inflammatory pain. Firstly, we found that both the expression of Neuregulin 1 (Nrg1) and phosphorylation of ErbB4 receptor were upregulated in DRG after inflammatory pain, implying the activation of ErbB4 in DRG. Using ErbB4-mutant mice, we found reduced pain sensitivity of mice when *ErbB4* gene expression was largely ablated; furthermore, ErbB4 deletion decreased the inflammatory pain hypersensitivity of either formalin- or CFA-induced mouse models. Moreover, the pain sensitivity was reduced in mice with specific deletion of ErbB4 on advillin-positive neurons within DRG. Importantly, pain hypersensitivity also decreased in *Advillin-Cre;ErbB4^-/-^* cKO mice after formalin- or CFA-induced inflammatory pain. Finally, gene quantification differential expression analysis, using RNAseq technology in combination with GO and KEGG enrichment analysis, suggested that calcium signaling pathway possibly mediated the roles of ErbB4 on DRG sensory neurons in inflammatory pain models. Together, these results indicate that ErbB4 on advillin-positive sensory neurons enhances inflammatory pain sensitivity, providing new clues towards the pathogenic mechanisms of inflammatory pain.

## Introduction

Inflammatory pain is a prevalent form of pathological pain in clinical practice with complex pathogenic factors [1-3]. It can result in negative emotions, such as anxiety and depression, leading to a significant reduction in patients’ quality of life [4, 5]. Current clinical treatments for inflammatory pain lack specificity [6-8] and mostly involve central analgesic drugs, which can cause several side effects [6, 9, 10]. Based on the gate control theory, the study of target-specific diagnosis and treatment of inflammatory pain needs to be more closely integrated with the peripheral nervous system [11, 12]. The stimulation of inflammatory pain is received by primary afferent neurons in the peripheral nervous system, which is the basis for pain signals to be transmitted to the brain [13]. The cell bodies of primary afferent neurons, which can detect noxious chemical, thermal, and mechanical stimuli, are located in dorsal root ganglion (DRG). Converging studies have shown that DRG plays a crucial role in processing and relaying sensory signals from nociceptor afferent fibers [14-16]. However, specific signaling pathways that exist in the DRG in the context of inflammatory pain still need to be further explored.

Recent studies have shown that multiple tyrosine kinases and their mediated signaling pathways can act on distinct populations of DRG neurons, such as Fyn and Trk [17-19]. Fyn regulates dendritic development of pyramidal neurons by phosphorylating CRMP1 at the Tyrosine 504 residue (Tyr504) in DRG neurons [19]. Co-activation of TrkA and TrkB signaling pathways has been demonstrated to reduce neuronal apoptosis and neurite loss in DRG [20]. As a member of the ErbB family of receptor tyrosine kinases, ErbB4 is mainly activated by the Neuregulin 1 (NRG1) and widely distributed in the central nervous systems [21]. ErbB4 regulates numerous physiological processes, including neuronal development and synaptic plasticity [22]. Recent studies have suggested that neurons expressing ErbB4 tyrosine kinases in the spinal cord can mediate their effects on heat sensation [23, 24]. However, the role of ErbB4 in DRG (key regulatory organ for transmitting pain and nociception signals) remains unclear so far [24, 25], and whether ErbB4 is directly involved in inflammatory pain through its action within DRG has not fully understood.

Here, we provide novel clues that ErbB4 within DRG participates in the regulation of inflammatory pain sensitivity. In this study, we explored the role of ErbB4 in DRG in two kinds of inflammatory pain models using ErbB4-mutant mice. Our findings showed that the phosphorylation of ErbB4 tyrosine kinase is upregulated in DRG after inflammatory pain that is induced by elevated NRG1 expression. Moreover, ablation of ErbB4 reduces inflammatory pain hypersensitivity in both formalin-induced and CFA-induced mouse models. Furthermore, specific ablation of ErbB4 from the advillin-positive sensory neurons decreased pain sensitivity in inflammatory pain models, that is probably mediated by the calcium signaling pathway. Thus, these findings underscore the contribution of ErbB4 in DRG neurons to inflammatory pain sensitivity. This work might help us to better understand the molecular cellular mechanisms underlying the pathogenesis of inflammatory pain and provide novel insights into therapeutic strategies alleviating pain.

## Materials and Methods

### Animals

We purchased C57BL/6J (2-4 months old) mice from the Beijing Viton Lihua Laboratory Animal Technology Co.LTD. Advillin-Cre, ErbB4-knockout [26], and ErbB4-floxed [27] mice were previously described. Using *Cre-loxP* technology, we crossed *Advillin-Cre* mice with *ErbB4^flox/flox^* mice to generate *ErbB4* conditional knockout (cKO) mice. As it is difficult to obtain *ErbB4^-/-^* homozygotes because of a very high lethality rate caused by poor development of their cardiovascular system, we mainly used heterozygotes in this study. We maintained the mice under SPF-grade animal room at room temperature in a 12h/12h day/night cycle with free access to food and water. Age-and gender-balanced littermates were randomly assigned to the experimental groups. We conducted behavioral tests were conducted on adult male mice aged 2-4 months. Tests were conducted during the day cycle, specifically from 9:00 am to 2:00 pm. The experimenters were blinded to both the genotypes and treatments of the mice. All experimental protocols were approved by the Animal Ethics Committee of Guangxi Medical University (Nanning, Guangxi, China).

### RNA extraction, cDNA synthesis, reverse-transcription PCR, and real-time PCR.

The L4-L6 DRGs were collected to detect the expression level of the indicated genes 24 hrs after formalin injection or at the indicated timepoint after CFA injection. Total RNA was extracted from mouse DRG using TRIzol reagent (Invitrogen, USA). Revert Aid First Strand cDNA Synthesis Kit (Thermo Scientific, USA) was used to reverse transcribe total RNA into cDNA and SYBR Green Master Mix (Vazyme, China) was used for real-time PCR. We used *Gapdh* as a control RNA. We used the same primers as those used for the RT-PCR. The primers used in the experiments: *Gapdh*, Forward primer: GGTTGTCTCCTGCGACTTCA, Reverse primer: CCACCACCCTGTTGCTGTAG; *ErbB4*, Forward primer: ACTACCCACACCTAGTT CGC, Reverse primer: CTGCCAGATCCCGATGAAC A; *Nrg1*, Forward primer: ATGTGCAAAGTGATCAGC AAG, Reverse primer: TGAGGACACATAGGGTCTTT.

### Western Blot

Tissues were homogenized by RIPA buffer containing protease inhibitors, followed by centrifugation to remove debris at 12,000 rpm for 20 min at 4°C. Protein concentrations were measured using the bicinchoninic acid protein assay kit and denatured by heating at 100°C for 10 min. The resulting protein samples were transferred to PVDF membranes from the SDS-PAGE gel and then were incubated in TBST containing 5% skim milk or 5% bovine serum albumin (BSA) for 1 h at room temperature before overnight primary antibody incubation at 4°C. Thereafter, the membranes were incubated with the secondary antibodies for 2 h. After incubation with enhanced chemiluminescence, immunoreactive bands were visualized using Bio-RAD automatic imaging system scanners. The intensity of immunoreactive bands was quantitated with ImageJ, with GAPDH (KC-5G4, 1:10000, Aksomics) as the loading control. For primary antibodies, we used mouse anti-Nrg1 (ab2369, 1:1000, Abcam), mouse anti-ErbB4 (MA1-861, 1:1000, Thermo Fisher Scientific), rabbit anti-p-ErbB4 (#4757, 1:200, Cell Signaling Technology); For sencondary antibodies, we used goat anti-rabbit (KC-RB-035, 1:10000, Aksomics) or goat anti-mouse (KC-MM-035); ditto HRP-conjugated secondary antibodies.

### Histology and Immunostaining

Mice were anesthetized by inhalation of isoflurane and perfused with saline followed by 4% paraformaldehyde (PFA). The L4-L6 DRGs were postfixed in 4% PFA for 10-12 h at 4°C and transferred to 15% and 30% sucrose in phosphate buffer for 10-12 h at 4°C. Samples were embedded in Optimal Cutting Temperature (OCT) medium (SAKURA, Japan). Frozen blocks were sliced with a cryostat (Leica, CM1950) to prepare 12 mm-thick slices. Slices were washed with 1 × phosphate buffered saline (PBS) and blocked with 1 × PBS containing 0.5% Triton-100 and 10% donkey serum for 1 h at room temperature, before overnight incubation of rabbit anti-ErbB4 antibody (0618, 1:200, recognizing residues 1108-1136 [28]) at 4°C. After washing in 1 × PBS three times, the slices were incubated in Alexa Fluor 488 conjugated donkey anti-rabbit IgG H&L (A21206, 1:500, Thermo Fisher Scientific) for 2 h at room temperature. After washing in 1 × PBS three times, the slices were incubated with DyLight™ 594 GSL IB4 isolectin (DL-1207-.5, 1:100, Vector Laboratories) after secondary antibody for overnight at 4?, and then were counterstained with Hoechst (#33342, 1:8000, Beyotime). The slices were only incubated in the second antibodies as a negative control. We visualized the samples using confocal laser scanning microscopy (Leica, Germany).

### RNAscope in situ Hybridization Technology Combined with Immunofluorescence Staining for Protein

We performed *in situ* hybridization (ISH) using the RNAscope^®^ Multiplex Fluorescent Reagent Kit v2 in combination with immunofluorescence, according to the manufacturer's integrated co-detection protocol (Advanced Cell Diagnostics, USA). The mice were anesthetized by inhalation of isoflurane and perfused with saline, followed by 4% PFA. The L4-L6 DRGs were postfixed in 4% PFA for 10-12 h at 4°C and transferred to 15% and 30% sucrose in phosphate buffer for 10-12 h at 4°C. Samples were embedded and then sliced with the cryostat to prepare 12 mm-thick frozen slices. The DRG slices were treated with Co-Detection Target Retrieval Reagents for 5 min at 100?, and then incubated with goat anti-CGRP antibody (ab36001, 1:200, Abcam) and mouse anti-NF200 antibody (MAB5266, 1:200, Millipore) for overnight at 4?. After washing in 1 ×PBS with Tween (PBST), protease plus reagent was added to each section until fully covered, the sections were incubated with the RNAscope Probe Mm-*Nrg1* (No.468841; Advanced Cell Diagnostics, USA) or RNAscope Probe Mm-*ErbB4* (No.318721; Advanced Cell Diagnostics, USA ) for 2 h at 40?. The control slices were incubated with RNAscope positive probe (No.321811; Advanced Cell Diagnostics, USA) or negative probe (No.321831; Advanced Cell Diagnostics, USA). The sections were then treated with RNAscope^®^ Multiplex FL v2 AMP reagents for signal amplification, and the HRP signal was developed according to the manufacturer’s protocol. Fluorescent labeling of RNAscope Probe was performed using Opal™ 620 dye (ASOP620; Akoya Biosciences) or Opal™ 570 dye (ASOP570; Akoya Biosciences). The sections were further labeled with Alexa Fluor 488 conjugated donkey anti-mouse IgG H&L (A21202, 1:500; Thermo Fisher Scientific) and Alexa Fluor 647 conjugated donkey anti-goat IgG H&L (A32849, 1:500; Thermo Fisher Scientific). Finally, they were mounted with an anti-fade mounting medium (Southern Biotech) and imaged using confocal laser scanning microscopy.

### Animal Modeling and Behavioral Tests

To induce acute or chronic inflammatory pain, we divided the mice into groups after being habituated in Perspex chambers with a wire mesh floor for at least 2 h. Subsequently, they were injected with 20 μL formalin or CFA into the bottom of the hind paws of the mice, while the vehicle group received an equal volume of normal saline[29]. In the pain-related behavior tests, the controls were from the littermates of the mutant mice. Before behavioral tests, the animals were subjected to habituation, which consisted of three trials of free exploration in the test apparatus, 30 min *per* day for three consecutive days.

To measure paw swelling induced by inflammatory pain, a marker line was drawn on the ankle joint of the left hind paw of each mouse before. The paw was then submerged in a 1.5 mL EP tube with liquid solution until the marker line was level with the liquid surface. The remaining liquid in the EP tube was aspirated using a pipette. The volume of the aspirated liquid was measured by adjusting the pipette scale, and the value obtained by subtracting the aspirated volume from 1.5 mL was recorded as the hind paw volume. The difference in paw volume was measured by subtracting the initial paw volume from the paw volume measured at each time point[30].

Von Frey test. To measure the mechanical pain sensitivity, we applied Electronic Von Frey filaments (No.38450; Ugo Basile, Italy) from the underside of the mesh to assess the paw withdrawal mechanical threshold. The filaments delivered continuous pressure with increasing intensity until the characteristic bending of the filament was observed. The pressure at which the mouse displayed the hind paw contraction reflex was recorded automatically, along with the time from the initial pressure to the maximum value. Each mouse was tested 10 times, with an interval of at least 30 seconds [31, 32].

Tail flick test. The mice were placed in a mouse immobilizer with their tails fully exposed outside the immobilizer, and the test was initiated when they were quiet. The instrument automatically stopped the stimulus when the mouse shook its tail in response. Each mouse was tested 10 times at 15 s intervals [33].

Cold plate test. The temperature of the metal plate of the apparatus was set at a constant temperature of 4°C and the animals were placed into the testing apparatus. The number of times the mice licked, violently shook, or quickly retracted their feet within five min was recorded. Each mouse underwent three 5-min tests, with a 15-min break between each test and an additional 60 seconds of adaptation time before the first test. The surface of the cold plate was thoroughly cleaned with an approved disinfectant before and after use [34].

Hot plate test. We placed the mice on a hot plate at a preset temperature of 55 ± 0.5°C. When the mice displayed escape behaviors such as licking, lifting, violent shaking, or jumping, the timing was immediately stopped. To avoid tissue damage, a cut-off time of 30 s was set. Each mouse was tested three times, each time at a 15-min interval, and the average of the paw withdrawal thermal latency results was used for subsequent statistical analysis [35].

Persistent Spontaneous Paw Reflex. For the formalin-induced acute inflammatory pain model, pain hypersensitivity was measured by spontaneous behaviors such as paw licking and flinching. After injection of 2% formalin into the left hind paw, the mice were immediately placed in a Perspex chamber with a wire mesh floor. Nociceptive behaviors were measured as licking, flicking, biting or shaking of the injected paw only. We recorded the mice’s behaviors at 5 min intervals for a duration of 60 min and divided into two phases, the first phase lasting 0-10 min and the second phase 10-60 min. And the late response after the pain stimuli can be subdivided into the IIA phase (10-40 min) and the IIB phase (40-60 min)[36, 37].

The behavioral devices were cleaned by 75% ethanol after each mouse completed tests. All the behavioral tests were performed double-blindly.

### RNA-sequencing

*Advillin-Cre;ErbB4*^-/-^ mice and wild-type mice were divided into four groups: vehicle-ctrl (control littermates receiving saline), vehicle-cKO (*Advillin-Cre;ErbB4*^-/-^ mice receiving saline), CFA-ctrl (control littermates receiving CFA), and CFA-cKO (*Advillin-Cre;ErbB4*^-/-^ mice receiving CFA). The RNA samples from each group were collected from the DRG of 3 mice, with 5-6 DRGs obtained from each mouse. cDNA library construction and sequencing were performed by high-throughput transcriptome sequencing at BGI (Beijing Genomics Institute) Corporation (Shenzhen, China). Differential expression genes were analyzed using BGI Dr. Tom 2.0. The gene ontology (GO) enrichment analysis and Kyoto Encyclopedia of Genes and Genomes (KEGG) analysis were performed online (www.bioinformatics.com.cn). The diagram of KEGG was visualized by Cytoscape_v3.9.1 software and polished by Adobe Illustrator CC 2019 software.

### Quantification and Statistical Analysis

For statistical analyses, the investigators were blinded to the genotypes and treatments of the animals. Shapiro-Wilk test is used to assess the normal distribution of all data. For normal distributed data, we used Student’s *t*-test; for non-normal data or small sample sizes, we used non-parametric alternative. For comparisons between multiple groups, we used one-way ANOVA with *post hoc* Dunnett’s tests. All statistical analyses were performed using SPSS 26.0 software. Data were presented as mean ± SEM, statistical significance was set at *P* < 0.05.

## RESULTS

### Inflammatory pain increases NRG1 expression and ErbB4 activation in DRG

**Figure 1.**
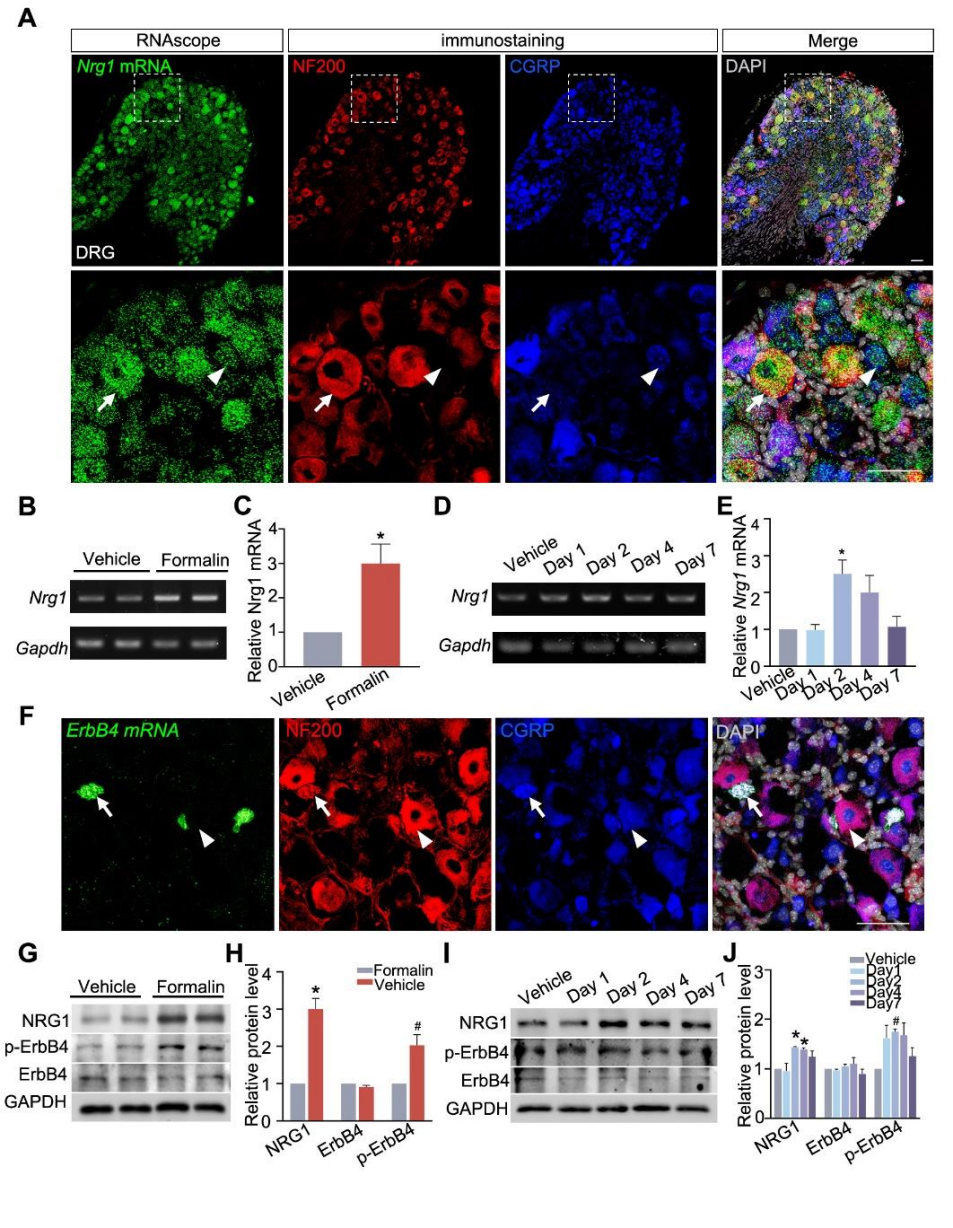
**Activation of NRG1-ErbB4 signaling in DRG after formalin- or CFA-induced inflammatory pain. (A)** The distribution of *Nrg1* mRNA in DRG detected by *in situ* hybridization via RNAscope in combination with immunostaining. NF200 and CGRP were used as protein markers for large- or small-diameter neurons, respectively. DAPI was used for counterstaining of the nucleus (gray). Dotted regions in the upper panels are shown at higher magnification below. Arrows and arrowheads indicate NF200+ neurons and CGRP+ neurons, respectively. Scale bar, 50μm. **(B-E)** PCR analysis of *Nrg1* mRNA expression in DRG after formalin- or CFA-induced inflammatory pain. **(B and D)** Representative bands of RT-PCR experiments; **(C and E)** semi-quantitative results of Real-time PCR experiments. *Gapdh* was used as an internal control. *n* = 3 samples *per* group. Each sample represents pooled DRGs of three mice. ^*^P = 0.037, Mann-Whitney U test, for formalin; ^*^P = 0.019, one-way ANOVA with *post hoc* Dunnett’s test, for CFA. **(F)** *ErbB4* expression revealed by RNAscope *in situ* hybridization in mouse DRG. Arrows and arrowheads indicate NF200+ neurons and CGRP+ neurons, respectively. Scale bar, 50μm. **(G-J)** Western blotting analysis of the indicated proteins in DRG after formalin- or CFA-induced models. GAPDH immunoblotting verified equal. **(G and I)** Representative blots; **(H and J)** statistical results. Each sample represents pooled DRGs of three mice. ^*^P_(NRG1)_ = 0.014, ^*^P_(NRG1-Day2)_ = 0.018, ^*^P_(NRG1-Day4)_ = 0.034; ^#^*P*_(p-ErbB4)_ = 0.014, ^#^*P*_(p-ErbB4-Day2)_ = 0.047; *n* = 4, Mann-Whitney U test, for formalin; *n* = 3, one-way ANOVA with *post hoc* Dunnett’s test, for CFA. n.s., not significant. Data were expressed as mean ± SEM.

As Nrg1 is the major ligand for ErbB4 tyrosine kinases [21], we conducted RNAscope *in situ hybridization* combined with compatible immunohistostaining at single-cell resolution, in order to detect the spatial distribution of *Nrg1* mRNA in DRG neurons. We observed *Nrg1* mRNA expressed by distinct-diameter neurons within DRG, including both large-diameter neurons expressing neurofilament 200 (NF200) and small-diameter neurons expressing calcitonin gene-related peptide (CGRP) (Fig. 1A). To test whether *Nrg1* transcription is responsive to pain activity, we detected the expression level of *Nrg1* mRNA by RT-PCR and real-time PCR, using acute inflammatory pain model induced by formalin (Fig. B-C). The expression level of *Nrg1* mRNA in DRG increased after acute inflammatory pain, at 2.99 ± 0.33-fold above the control level (Fig. 1B and C). To determine whether *Nrg1* upregulation is a general phenomenon in DRG, we further used chronic inflammatory pain model induced by CFA. As observed in the formalin model, CFA-induced pain activity also caused an increase in *Nrg1* mRNA expression with 2.50 ± 0.38-fold above the vehicle level 2 days later (Fig. 1D and E). To confirm the expression of ErbB4 in DRG, we continued to perform RNAscope detection and found scatted distribution of ErbB4 in small-diameter DRG neurons (Fig. 1F). Subsequently, we further determined whether ErbB4 is activated by elevated NRG1 expression in DRG by measuring its phosphorylation (Fig. 1G-J). As shown in Figure 1G and H, the expression of phosphorylated ErbB4 (p-ErbB4) increased 2.03 ± 0.28-fold in DRG, 24 hrs after formalin injection. In chronic inflammatory pain model induced by CFA, the expression of p-ErbB4 also increased 1.75 ± 0.06-fold on the second day (Fig. 1I and J). Of note, the total ErbB4 protein level remained unchanged after inflammatory pain in both models (Fig. 1G-J). Thus, these results suggest that inflammatory pain induces the activation of ErbB4 phosphorylation in mouse DRG.

**Figure 2.**
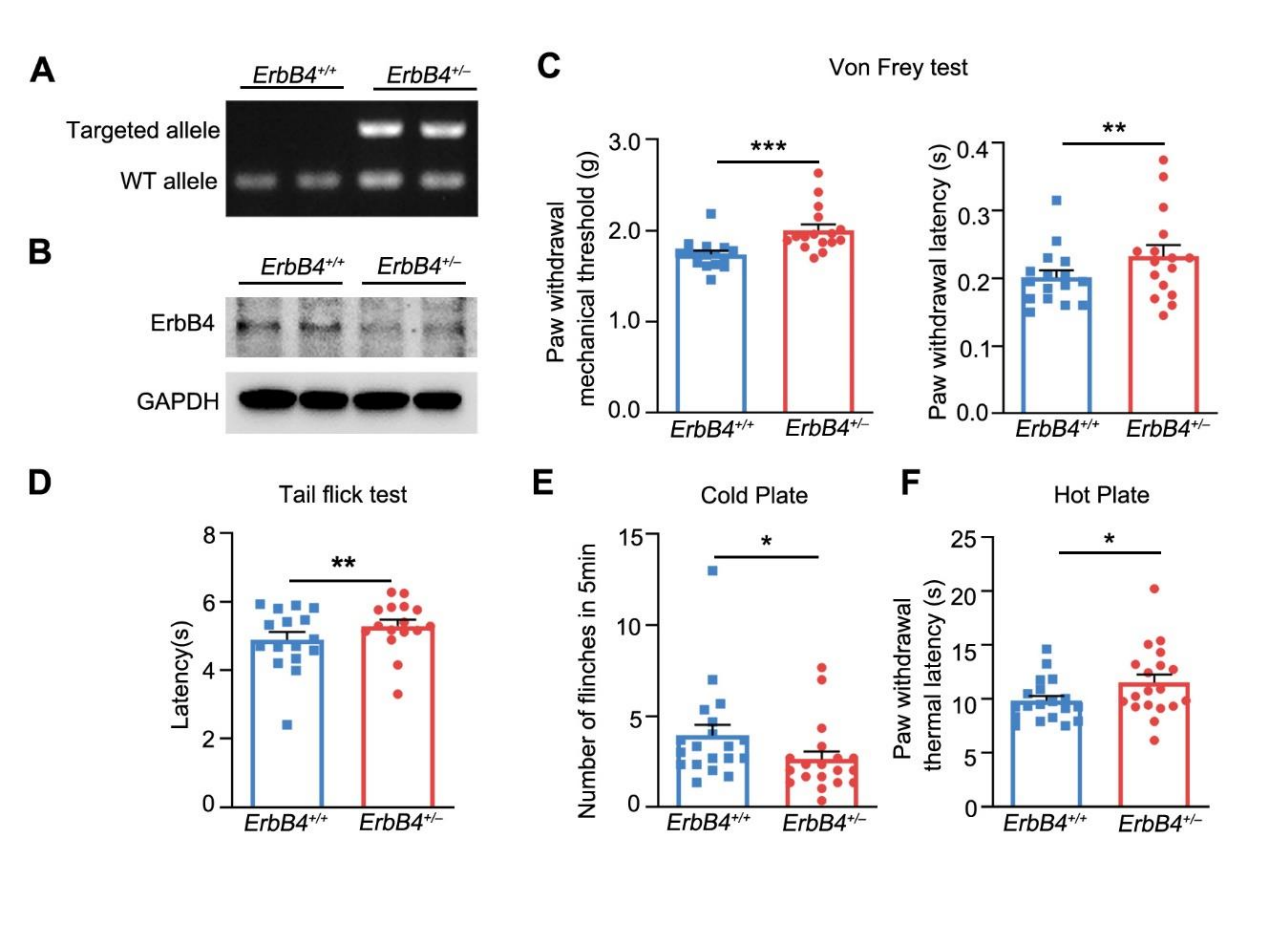
***ErbB4*-mutant mice showed reduced pain sensitivity under physiological condition. (A)** Genotyping of *ErbB4^+/-^* mice. **(B)** Representative blots of ErbB4 expression in heterozygous DRG. **(C)** Increased mechanical threshold (left) and latency (right) in Von Frey test in *ErbB4^+/-^* mice. *n* = 16 *per* group. Left, ^***^P = 0.0001; Right, ^**^P = 0.003, two-tailed paired student’s *t*-test. **(D)** Increased response latency in tail-flick test in *ErbB4^+/-^* mice. *n* = 16 *per* group. ^**^P = 0.009, two-tailed paired student’s *t*-test. **(E)** Reduced number of flinches in cold plate test in *ErbB4^+/-^* mice. *n* = 19 *per* group. ^*^P = 0.038, two-tailed paired student’s *t*-test. **(F)** Increased thermal latency in hot plate test in *ErbB4^+/-^* mice. *n* = 19 *per* group. ^*^P = 0.021, two-tailed paired student’s *t*-test. Data were expressed as mean ± SEM.

**Figure 3.**
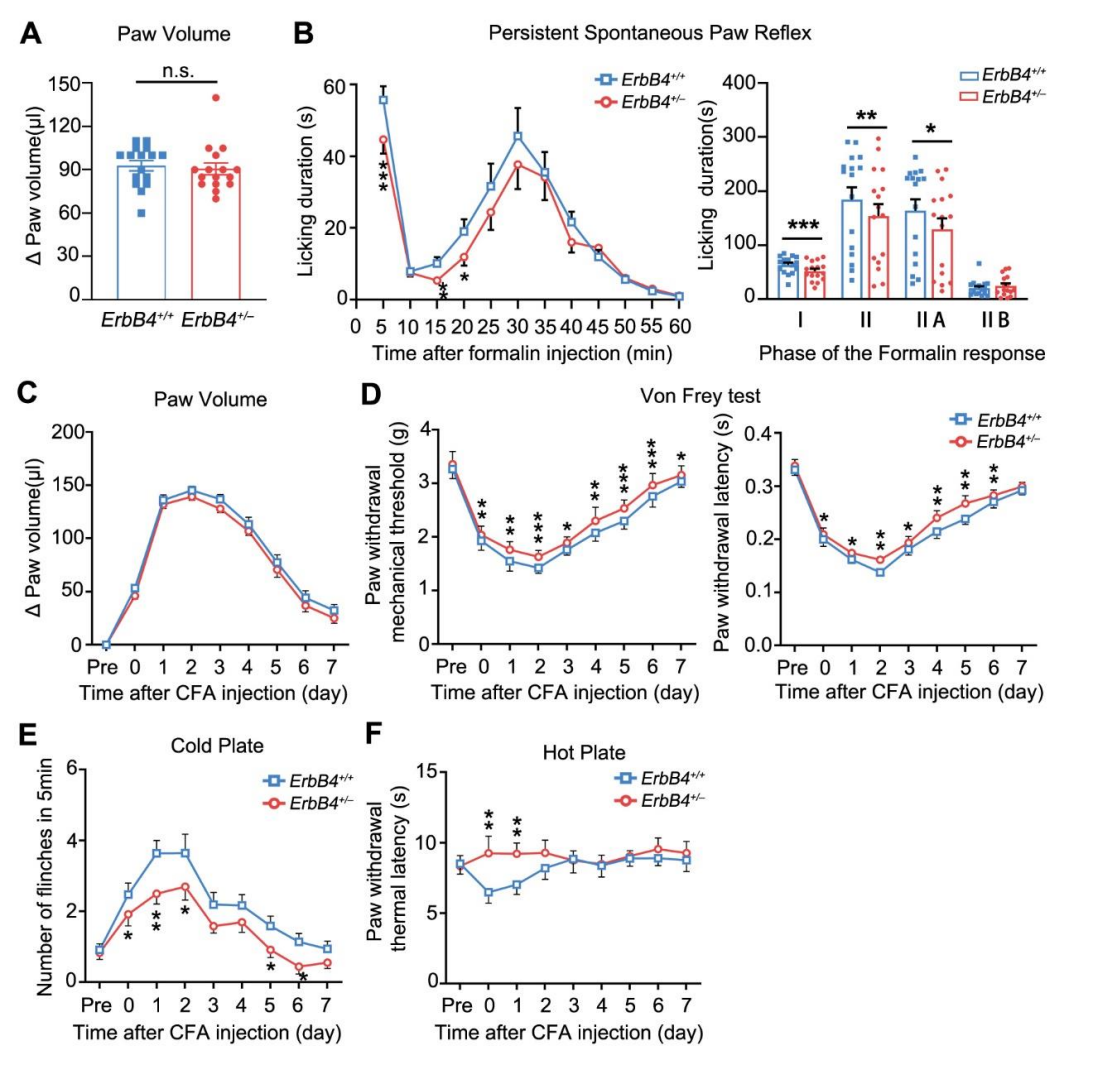
***ErbB4* ablation decreased pain hypersensitivity induced by formalin or CFA. (A)** No significant difference in paw volume between mutant and wild-type mice after formalin-induced acute inflammatory pain. *n* = 19 *per* group. *P* = 0.512, two-tailed paired student’s *t*-test. **(B)** The biphasic plot of formalin-induced pain in *ErbB4*^+/-^ mice. Left, time course of formalin-induced nociceptive responses. ^***^P_(5min)_ = 0.0000, ^**^P_(15min)_ = 0.002, ^*^P_(20min)_ = 0.013, two-tailed paired student’s *t*-test. Right, phase I and phase II summed paw licking behavior after formalin-induced pain. ^***^P_(I)_ = 0.0000, ^**^P_(II)_ = 0.005, ^**^P_(IIA)_ = 0.001, two-tailed paired student’s *t*-test. *n* = 16 *per* group. **(C)** Time course of paw volume between mutant and wild-type mice after CFA-induced chronic inflammatory pain. *n* = 11 *per* group. **(D)** Time course of mechanical threshold (left) and latency (right) in Von Frey test in *ErbB4*^+/-^ mice after CFA induction. Left, ^**^P_(day0)_ = 0.004, ^***^P_(day1)_ = 0.0002, ^***^P_(day2)_ = 0.0000, ^**^P_(day3)_ = 0.001, ^**^P_(day4)_ = 0.003, ^***^P_(day5)_ = 0.0002, ^***^P_(day6)_ = 0.0000, ^*^P_(day7)_ = 0.016. Right, ^*^P_(day0)_ = 0.043, ^*^P_(day1)_ = 0.019, ^**^P_(day2)_ = 0.003, ^*^P_(day3)_ = 0.044, ^**^P_(day4)_ = 0.004, ^**^P_(day5)_ = 0.005, ^**^P_(day6)_ = 0.006, two-tailed paired student’s *t*-test. **(E)** Flinches number of *ErbB4*^+/-^ mice in 5 min of cold plate test every day after CFA injection. ^*^P_(day0)_ = 0.041, ^*^P_(day1)_ = 0.008, ^*^P_(day2)_ = 0.049, ^*^P_(day5)_ = 0.03, ^*^P_(day6)_ = 0.026, two-tailed paired student’s *t*-test. **(F)** Time course of thermal latency as assessed by hot plate test after CFA injection in *ErbB4^+/-^* mice. ^**^P_(day0)_ = 0.007, ^**^P_(day1)_ = 0.009, two-tailed paired student’s *t*-test. Data were expressed as mean ± SEM.

### Ablation of ErbB4 attenuates pain sensitivity

To determine whether ErbB4 is involved in regulating pain sensitivity, we performed a series of experiments to evaluate pain behaviors in ErbB4 knockout mice. Due to the very high lethality of *ErbB4*^-/-^ homozygotes, we here mainly used heterozygous mutant mice with large-degree ablation of ErbB4 protein (Fig. 2A and B). To observe mechanical stimulation-induced pain sensitivity under physiological condition, we performed Von Frey test and found that *ErbB4*^+/-^ mice had a higher paw withdrawal mechanical threshold (Fig. 2C, left) and latency (Fig. 2C, right). Moreover, *ErbB4*^+/-^ mice were less sensitive to photo-thermal stimulation than the wild-type littermates, as revealed by tail flicking tests (Fig. 2D). Cold plate tests showed that *ErbB4*^+/-^ mice showed less spontaneous paw licking, shaking, or rapid paw shrinkage within 5 min than control mice (Fig. 2E); while hot plate tests showed that mutant mice had a higher thermal pain threshold than control mice (Fig. 2F).

Next, we further determined the involvement of ErbB4 kinase in pathogenesis of inflammatory pain sensation using both acute and chronic pain models. There was no significant difference in formalin-induced paw swelling between the two genotypes (Fig. 3A). However, biphasic plot showed *ErbB4*^+/-^ had decreased licking duration within 60 minutes after acute inflammatory pain (Fig. 3B, left), especially in Phase I and Phase IIA (Fig. 3B, right).

Furthermore, we observed changes in paw swelling (Fig. 3C) and pain-related behavioral manifestations within 7 days after CFA injection, using *ErbB4*^+/-^ mice and their control littermates. As shown in Figure 3D, *ErbB4*^+/-^ mice exhibited a higher paw withdrawal mechanical threshold compared to their control littermates (left), with a prolonged response time (right) during 1-6-day period following the induction of chronic inflammatory pain. Interestingly, the number of flinches on the cold plate was significantly reduced in the *ErbB4*^+/-^ mice compared to the wild-type mice, with the most significant decrease observed in 1-2 days after CFA injection (Fig. 3E). In hot plate test, *ErbB4*^+/-^ mice displayed a delayed paw withdrawal thermal latency than the wild-type littermates (Fig. 3F). Together, the above results clearly show that ablation of ErbB4 decreases the pain sensitivity and increases the latency of mice in response to inflammatory pain, suggesting an important role of ErbB4 kinases in regulating inflammatory pain.

**Figure 4.**
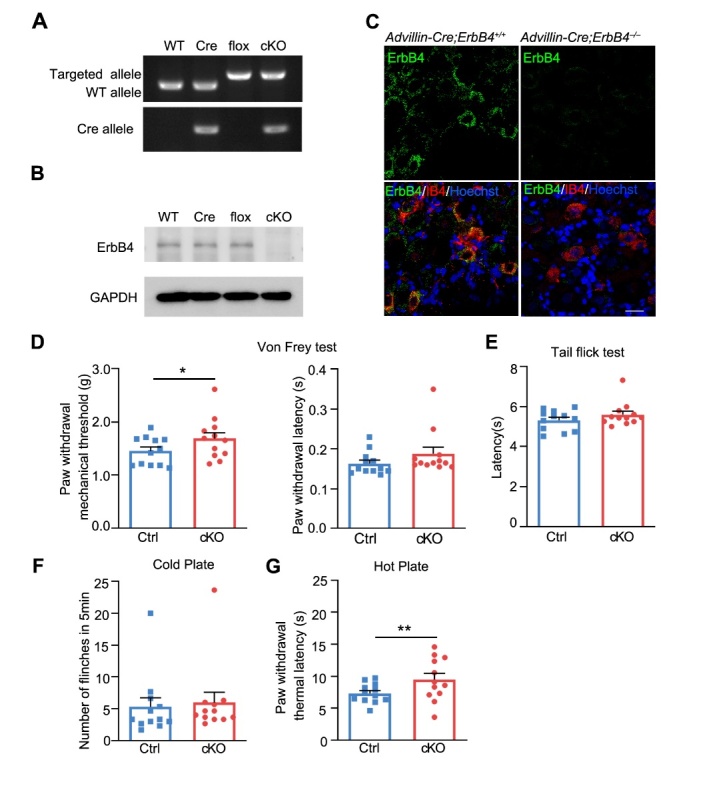
**Pain sensitivity was reduced by specific deletion of ErbB4 in Advillin-positive DRG neurons under physiological condition. (A)** Genotyping of *Advillin-Cre;ErbB4^-/-^* cKO mice. **(B)** Western blotting analysis of ErbB4 expression in *Advillin-Cre;ErbB4^-/-^* mice and their control littermates. **(C)** Immunofluorescence staining showed no detection of ErbB4 protein in small-diameter neurons of DRG in *Advillin-Cre;ErbB4^-/-^* mice. Scale bar, 25 μm. **(D)** Increased mechanical threshold (left) and latency (right) in Von Frey test in cKO mice. *n* = 12 *per* group. ^*^P = 0.011, two-tailed paired student’s *t*-test. **(E)** Increased response latency in tail flick test in mice. **(F)** Cold plate test in *Advillin-Cre; ErbB4^-/-^* mice. **(G)** Increased thermal latency in hot plate test in cKO mice. ^**^P = 0.009, two-tailed paired student’s *t*-test. Data are expressed as mean ± SEM.

**Figure 5.**
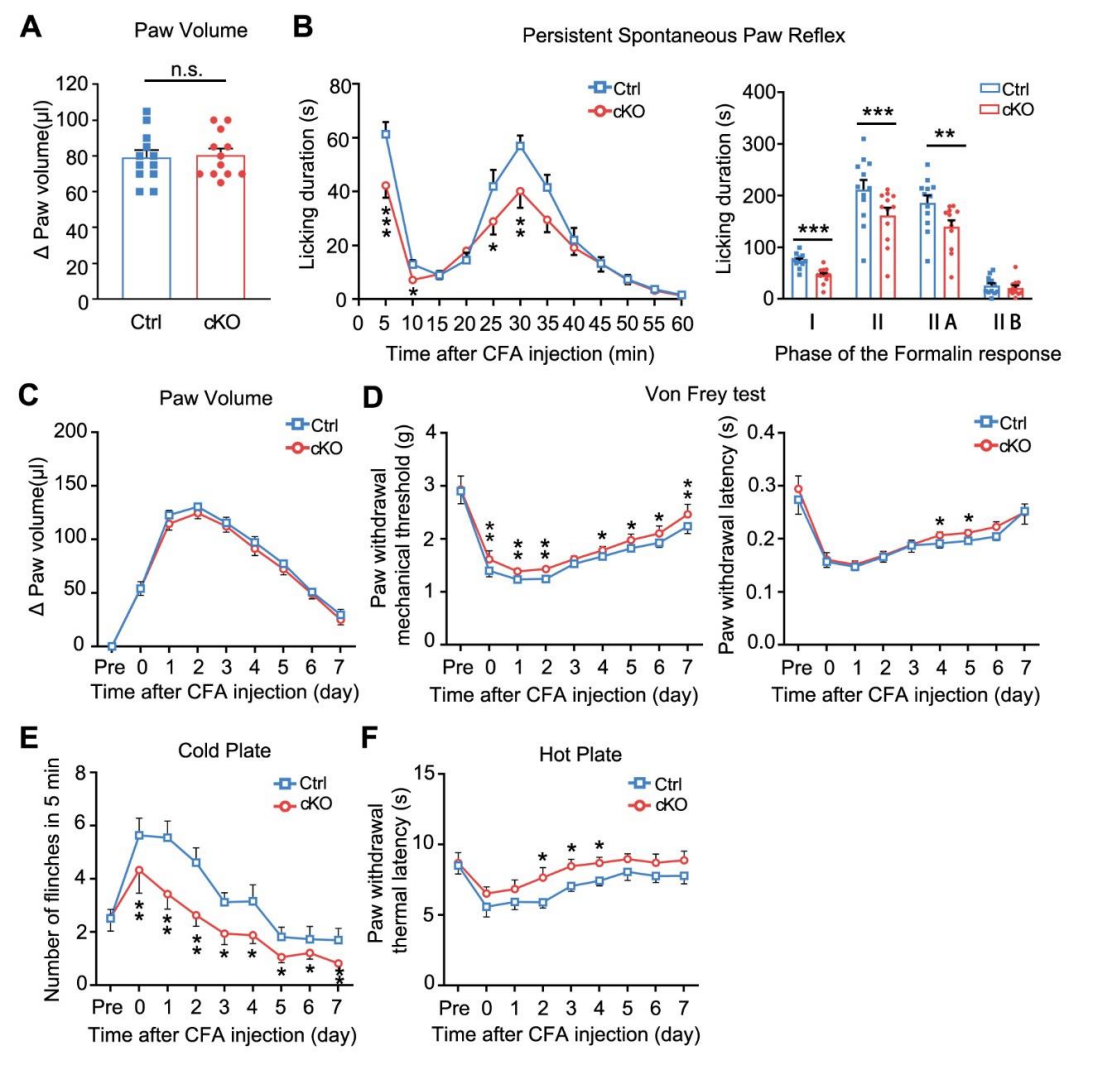
**Pain hypersensitivity decreased in *Advillin-Cre; ErbB4^-/-^* mice after formalin- or CFA-induced inflammatory pain. (A)** No significant difference in paw volume between cKO mice and their control littermates after formalin-induced acute inflammatory pain. *n* = 12 *per* group. *P* = 0.623, two-tailed paired student’s *t*-test. **(B)** The biphasic plot of formalin-induced pain in *Advillin-Cre; ErbB4^-/-^* mice. Left, time course of formalin-induced nociceptive responses. ^***^P_(5min)_ = 0.0005, ^*^P_(10min)_ = 0.019, ^*^P_(20min)_ = 0.02, ^**^P_(35min)_ = 0.006. Right, phase I and phase II summed paw licking behavior after formalin-induced pain. ^***^P_(I)_ = 0.0001, *^**^P_(II)_ = 0.0000, ^**^P_(IIA)_ = 0.003, two-tailed paired student’s *t*-test. **(C)** Time course of paw volume between cKO mice and their control littermates after CFA-induced chronic inflammatory pain. *n* = 11 *per* group. **(D)** Time course of mechanical threshold (left) and latency (right) in Von Frey test in cKO mice after CFA induction. Left, ^**^P_(day0)_ = 0.003, ^**^P_(day1)_ = 0.003, ^**^P_(day2)_ = 0.002, ^*^P_(day4)_ = 0.016, ^*^P_(day5)_ = 0.036, ^*^P_(day6)_ = 0.023, ^**^P_(day7)_ = 0.005. Right, ^*^P_(day4)_ = 0.048, ^*^P_(day5)_ = 0.029, two-tailed paired student’s *t*-test. **(E)** Flinches number of cKO mice in 5min of cold plate test every day after CFA injection. ^*^P_(day0)_ = 0.034, ^**^P_(day1)_ = 0.006, ^**^P_(day2)_ = 0.007,^*^P_(day3)_ = 0.049, ^*^P_(day4)_ = 0.015, ^*^P_(day7)_ = 0.0362. **(F)** Time course of thermal latency as assessed by hot plate test after CFA injection in cKO mice.^**^P_(day2)_ = 0.001,^*^P_(day2)_ = 0.023, ^*^P_(day4)_ = 0.026, two-tailed paired student’s *t*-test. Data were expressed as mean ± SEM.

### ErbB4 in advillin-positive neurons affects inflammatory pain hypersensitivity

As shown before, our results have shown that ErbB4 is predominantly expressed on small-diameter DRG neurons which convey the nociceptive information [38] (Fig. 2A). To investigate the specific role of ErbB4 in small-diameter DRG neurons, we generated *Advillin-Cre;ErbB4*^-/-^ mice which selectively lack ErbB4 in advillin-positive peripheral sensory neurons of DRG (Fig. 4A). Both western-blot analysis and immunofluorescence staining further confirmed the efficacy of conditional knockout of *ErbB4* gene in advillin-positive neurons (Fig. 4B and C). Behavioral experimental results showed that *Advillin-Cre;ErbB4*^-/-^ mice had a higher paw withdrawal mechanical threshold in response to Von Frey filament stimulation than the control littermates (Fig. 4D). Importantly, although we detected no statistical difference in tail flick and cold plate tests, *Advillin-Cre;ErbB4*^-/-^ mice exhibited a longer paw withdrawal thermal latency on the hot plate than the control mice (Fig. 4G). To further investigate the effect of ErbB4 in advillin-positive neurons on inflammatory pain sensitivity, we examined the degree of paw volume swelling and behavioral performance of *Advillin-Cre;ErbB4*^-/-^ mice and their control littermates following formalin injection (Fig. 5A). Biphasic plot showed that *Advillin-Cre;ErbB4*^-/-^ mice had decreased duration of the licking at the phase I and IIA, compared with the control mice (Fig. 5B).

**Figure 6.**
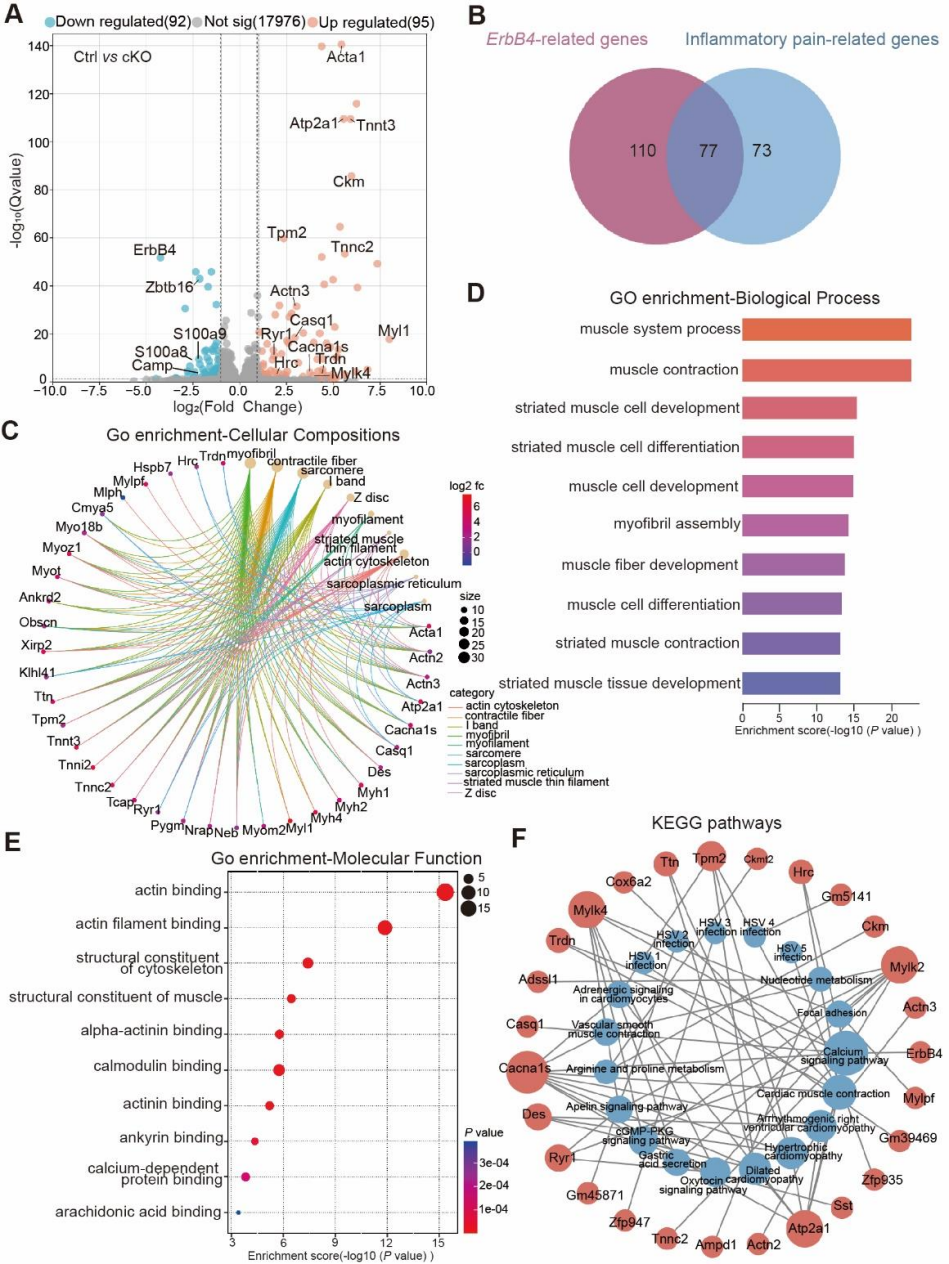
**Identification of key genes involved in inflammatory pain in *Advillin-Cre;ErbB4^-/-^*mouse DRG using transcriptomic data analysis. (A)** Volcano plot of differentially expressed genes between cKO and WT DRGs. Red dots represent upregulated genes, blue dots represent downregulated genes, and gray dots represent genes that were not differentially expressed (*P* < 0.05, |logFC| > 1.0). **(B)** Venn diagram showed the intersecting part in ErbB4-related genes and inflammatory pain-related genes. **(C)** Enriched gene ontology (GO) terms for cellular compositions (CCs) associated with the related genes. **(D)** Enriched GO terms for biological process (BP) associated with the related genes. **(E)** Enriched GO terms for molecular function (MF) associated with the related genes. **(F)** Enriched KEGG pathways for the related genes.

We also evaluated pain-related behaviors in CFA-induced chronic inflammatory pain on *Advillin-Cre;ErbB4*^-/-^ mice (Fig. 5C). Strikingly, we observed increased paw withdrawal mechanical threshold and latency in cKO mice (Fig. 5D). Both cold plate test and hot plate test revealed that the number of flinches decreased while the latency of paw withdrawal increased in cKO mice after induction of chronic inflammatory pain (Fig. 5E and F). Taken together, these results suggest that advillin-positive neurons mediated the effect of ErbB4 on inflammatory pain.

### Calcium signaling pathway possibly mediated the role of ErbB4 in pain

To explore the molecular mechanism by which ErbB4 affects the sensitivity of inflammatory pain, we then used RNAseq technology to perform high-throughput sequencing on the samples. Compared with the control mice, 187 differential genes related to ErbB4 were detected in the *Advillin-Cre;ErbB4*^-/-^ mice using a fold change of 2 and a p-value of 0.05 as cutoff values, of which 95 were upregulated and 92 were downregulated (Fig. 6A). Venn diagram showed that there are 77 genes related to both ErbB4 kinases and inflammatory pain (Fig. 6B). The 77 differential genes were annotated into cell composition, main biological processes and molecular function in the GO[39] function classification analysis (Fig. 6C-E). The 77 differential genes and *ErbB4* were then enriched into 19 different KEGG pathways[40], including the calcium signaling pathway, cardiac muscle contraction, etc. (Fig. 6F). KEEG enrichment results showed that *ErbB4* was closely related to calcium signaling pathway containing *Atp2a1, Cacna1s, Casq1, Hrc, Ryr1, Tnnc2, Mylk2, Mylk4* and *Trdn*, etc.

## DISCUSSION

In this work, we provide evidence that ErbB4 in DRG sensory neurons contributes to inflammatory pain sensitivity in mice. First, we observed an increase in the phosphorylation of ErbB4 in DRG after both acute and chronic inflammatory pain, indicating the activation of ErbB4 by inflammatory pain. Second, our observations revealed that the *ErbB4*^+/-^ mice have decreased pain sensitivity under physiological condition, as well as weaker hypersensitivity in inflammatory pain models than the control littermates. Third, similar behavioral phenotypes were observed in mice with conditional knockout of the ErbB4 gene in advillin-positive neurons of DRG. To our knowledge, this is the first evidence for the effect of ErbB4 tyrosine kinases in DRG on pain sensitivity in inflammatory pain models. Additionally, using GO and KEGG enrichment analysis, we found that calcium signaling pathway is involved in the regulation of inflammatory pain by ErbB4 in DRG. Together, these results indicate the involvement of ErbB4 in regulating inflammatory pain hypersensitivity that is mediated by advillin-positive sensory neurons.

DRG is a key structure for sensory conduction and regulation, playing an important role in transmission and integration processes of various nociceptive information [41, 42]. The subtypes of DRG neurons exhibit high heterogeneity. Small-diameter DRG neurons transmit harmful information via unmyelinated axons (C-fibers) and thinly myelinated axons (AδFibers). Large-diameter DRG neurons transmit mechanoreceptive and proprioceptive signals via thickly myelinated afferents [38]. Recent studies found that unique characteristics including selective somatic organization and high permeability blood vessels make the DRG an ideal target for neuromodulation [43]. It has recently been shown that epidermal growth factor receptor (EGFR) contributes to the pathogenesis of pain[44]. As a member of the EGFR family, ErbB4 in the dorsal horn of the spinal cord have been shown to be involved in heat hypersensitivity in inflammatory and neuropathic pain [23]. While previous studies have only focused on the effect of ErbB4 in spinal cord, our work here provides direct evidence for the role of ErbB4 within DRG in pathogenesis of inflammatory pain. Actually, ErbB4 was only found to be limited in a small proportion of DRG neurons in this study (Fig. 1F), in consistence with previous reports about low expression of ErbB4 in DRG homogenate [24, 45]. Interestingly, selective genetic ablation of ErbB4 in advillin-positive neurons decreased the pain sensitivity under physiological condition and inflammatory pain hypersensitivity in both kinds of mouse models, as evidenced by Von Frey test and hot plate test, indicating the involvement of ErbB4-expressing DRG sensory neurons in pain sensation (Fig. 4 and 5).

In this study, NRG1 is found to be enriched in DRG and upregulated by inflammatory pain activity (Fig. 1A, G-J). NRG1 can bind to multiple ErbB transmembrane kinases in DRG, including not only ErbB4 but also ErbB2 and ErbB3 [23], which are possibly involved in peripheral nerve injury [45]. Herein, whether other downstream pathways of NRG1 signaling participate in pathogenesis of pain sensation needs to be further determined. Besides, other Nrg1 binding proteins like Limk1 are also highly expressed in DRG and have been reported to modulate inflammatory pain [46]. Thus, there might exist a Nrg1-LIMK axis driving pathogenesis of inflammatory pain [47]. Additionally, there might be difference in developmental compensation between ErbB4 null mutant mice and the cKO ones. These above factors might explain the mild effect of ErbB4 cKO on pain sensation (Fig. 5). Nevertheless, ErbB4 on advillin-positive DRG neurons maybe act as a potential modulating targets for inflammatory pain.

During the pathogenesis of inflammatory pain, a large number of cellular cascades participate in the regulation of pain sensation, including calcium signaling pathway [48, 49]. In our study, comprehensive transcriptome analysis of *Advillin-Cre;ErbB4*^-/-^ mice identified 77 key genes possibly linked to the relationship between ErbB4 and inflammatory pain (Fig. 6A). Among them, KEGG analysis further revealed that *Atp2a1, Cacna1s, Casq1, Hrc, Ryr1, Tnnc2, Mylk2, Mylk4*, and *Trdn*, which probably participate in regulating intracellular calcium concentration through the calcium signaling pathway to modulate pain sensitivity [50-52], might mediate the effect of ErbB4 on inflammatory pain sensation (Fig. 6F). These molecules served as possible molecular mechanisms underlying the pathogenesis of inflammatory pain in *Advillin-Cre;ErbB4*^-/-^ mice. Further molecular biology experiments are required to validate the specific role of [the proteins/genes] in the relationship between ErbB4 and inflammatory pain.

In conclusion, ErbB4 on advillin-positive DRG sensory neurons contributes to pain hypersensitivity induced by pro-inflammatory factor, that is probably mediated by the calcium signaling pathway. Other underlying molecular cellular mechanisms need to be further explored in the future. Nevertheless, the present study provides useful clues towards the pathogenic mechanisms of inflammatory pain and help us to develop potential therapeutic strategies against it.
